# Reduced adenosine diphosphate sensitivity in skeletal muscle mitochondria increases reactive oxygen species production in mouse models of aging and oxidative stress but not denervation

**DOI:** 10.1002/rco2.29

**Published:** 2020-12-28

**Authors:** Gavin Pharaoh, Jacob Brown, Rojina Ranjit, Zoltan Ungvari, Holly Van Remmen

**Affiliations:** 1 Physiology Department, University of Oklahoma Health Sciences Center, Oklahoma City, OK, USA; 2 Aging & Metabolism Research Program, Oklahoma Medical Research Foundation, Oklahoma City, OK, USA; 3 Reynolds Oklahoma Center on Aging, University of Oklahoma Health Sciences Center, Oklahoma City, OK, USA; 4 Center for Geroscience and Healthy Brain Aging, Department of Biochemistry and Molecular Biology, University of Oklahoma Health Sciences Center, Oklahoma City, OK, USA; 5 Oklahoma Center for Neuroscience, University of Oklahoma Health Sciences Center, Oklahoma City, OK, USA; 6 Oklahoma City VA Medical Center, Oklahoma City, OK, USA

**Keywords:** Sarcopenia, Reactive oxygen species (ROS) production, Mitochondria, ADP sensitivity, Oroboros O2k respirometry and fluorometry

## Abstract

**Background:**

Mitochondrial bioenergetics are sensitive to adenosine diphosphate (ADP) concentration. Reactive oxygen species (ROS) production and respiration [oxygen consumption rate (OCR)] are altered at physiological ADP concentrations (i.e. ADP insensitivity) in aged human muscle. Here, we investigate ADP sensitivity in mouse muscle mitochondria.

**Methods:**

We measured OCR and ROS production in permeabilized gastrocnemius fibres using an ADP titration protocol and the Oroboros O2k respirometer and fluorometer. We measured changes in ADP sensitivity in muscle from mice at different ages, after sciatic nerve transection (denervation), and in response to increased oxidative stress (*Sod1*^−/−^ mice). Further, we asked whether the mitochondrial-targeted peptide SS-31 can modulate ADP insensitivity and contractile function in the *Sod1*^−/−^ mouse model.

**Results:**

Reduced ADP sensitivity is associated with increases in mitochondrial ROS production in aged (62%) and *Sod1*^−/−^ (33%) mice. The maximal capacity to produce ROS does not increase with age, and there is no effect of age on ADP sensitivity for OCR in mouse gastrocnemii. Denervation does not induce ADP insensitivity for either ROS generation or OCR. Treatment of *Sod1*^−/−^ mice with SS-31 increases ADP sensitivity for both OCR and ROS, decreases maximal ROS production (^~^40%), and improves resistance to muscle fatigue.

**Conclusions:**

Adenosine diphosphate sensitivity for ROS production decreases in aged mouse gastrocnemius muscle fibres, although aged mice do not exhibit a difference in OCR. Denervation does not induce ADP insensitivity; however, insensitivity to ADP is induced in a model of oxidative stress. ADP insensitivity could contribute to muscle fatigue, and SS-31 may be the first drug capable of targeting this aging phenotype.

## Introduction

The loss of skeletal muscle mass and strength with age (sarcopenia) is a complex process with many factors contributing to its progression. Sarcopenia affects up to 50% of people over age 80.^1^ Age-related skeletal muscle atrophy is a primary factor in decreased quality of life and a costly healthcare burden.^2,3^ The U.S. Food and Drug Administration has not approved any pharmaceutical treatments for sarcopenia. Mitochondrial dysfunction is one of the hallmarks of aging and plays a prominent role in skeletal muscle aging due to high energetic demands.^4,5^ Muscle strength declines more rapidly with age than muscle mass.^6^ Mitochondria are likely to contribute to the decline in muscle strength due to their role in the production of energy required for contraction as well as for the production of reactive oxygen species (ROS) that damages contractile machinery and decreases efficiency.^5^ While it is clear that mitochondria undergo age-related changes in function, how energy production and ROS production change in muscle has been controversial in the field. Multiple studies have found no change in ROS or energy production in aged human skeletal muscle.^7,8^ This controversy is likely due to the variety of methods used to measure mitochondrial energy and ROS production as well as the variability in mitochondrial content between fibre types, individual muscles, and species.^9–11^

The recent study by Holloway *et al.* provided a methodological advancement for measuring respiratory capabilities and ROS production in skeletal muscle that has helped elucidate age-related changes.^12^ This method uses permeabilized muscle fibres, which more closely model the function of mitochondria *in vivo* than isolated mitochondria by maintaining the complex mitochondrial networks found in skeletal muscle.^13^ The primary advancement of their method was to titrate adenosine diphosphate (ADP) in the presence of mitochondrial substrates in order to measure mitochondrial respiration and ROS production at physiological ADP levels.^12^ Free ADP concentrations in the mouse gastrocnemius muscle range from ^~^20 μM free ADP at rest to ^~^165 μM ADP during contraction, while many respirometry experiments use ADP concentrations in the millimolar range.^14^ Previous methods measured ROS production under a variety of non-physiological conditions including in the presence of mitochondrial substrates but absence of ADP, in the presence of supraphysiological ADP concentrations, or in the presence of electron transport system (ETS) inhibitors.^12^ ADP concentration is a critical component in measuring mitochondrial function, because increasing concentrations of ADP result in decreased ROS production and increased oxygen consumption rate (OCR).^12^ Experiments using supraphysiological ADP concentrations have found no difference in ROS production with age.^7^ Using the method described in Holloway *et al*. in human *vastus lateralis* biopsies, the authors established several key findings on age-related changes in muscle mitochondrial function. Neither respiratory capacity nor the maximum capacity to produce ROS increases or decreases with age. However, sensitivity to ADP stimulation decreases at physiologically relevant ADP concentrations in aged human muscle (ADP insensitivity). This results in an increase in ROS production and decrease in OCR (and ATP production) at these physiologically relevant concentrations. Together, these phenotypes result in an increase in electron leak to ROS production instead of ATP production.^12^

These findings raised many issues for the field. One of the most pertinent is whether aged mouse skeletal muscle replicates this phenotype. The goal of this study was to establish whether mice phenocopy this facet of human aging and sarcopenia. This finding will provide a platform for intervention testing to target ADP insensitivity and to study how it affects contractile properties. To address this issue, we studied ADP sensitivity in muscle fibres from young, middle-aged, and old mice. As oxidative stress and loss of innervation are associated with age-related muscle atrophy, we also examined if loss of innervation or oxidative stress can induce mitochondrial ADP insensitivity using two other atrophy models, sciatic nerve transection (denervation) and *Sod1*^−/−^ mice (oxidative stress). *Sod1*^−/−^ mice are a model of accelerated sarcopenia characterized by a progressive loss of neuromuscular innervation, muscle mitochondrial dysfunction, oxidative stress, and muscle atrophy.^15–18^ We also performed an intervention study in the *Sod1*^−/−^ mice using treatment with SS-31 (also known as Elamipretide or Bendavia), a mitochondrial-targeted peptide that has been shown to improve muscle mitochondrial function.^19,20^ With these experiments, we establish that aged and *Sod1*^−/−^ mice exhibit mitochondrial ADP insensitivity and produce increased ROS at physiological ADP concentrations. Surprisingly, denervated muscle does not exhibit ADP insensitivity, which suggests that ADP insensitivity is not driven by a loss of neuromuscular innervation. Treatment of *Sod1*^−/−^ mice with the mitochondrial-targeted peptide SS-31 increases ADP sensitivity and decreases muscle fatigue, providing evidence for the role of mitochondrial ADP insensitivity in contractile dysfunction.

## Results

### Sensitivity to adenosine diphosphate decreases in aging mouse skeletal muscle

**Figure 1:**
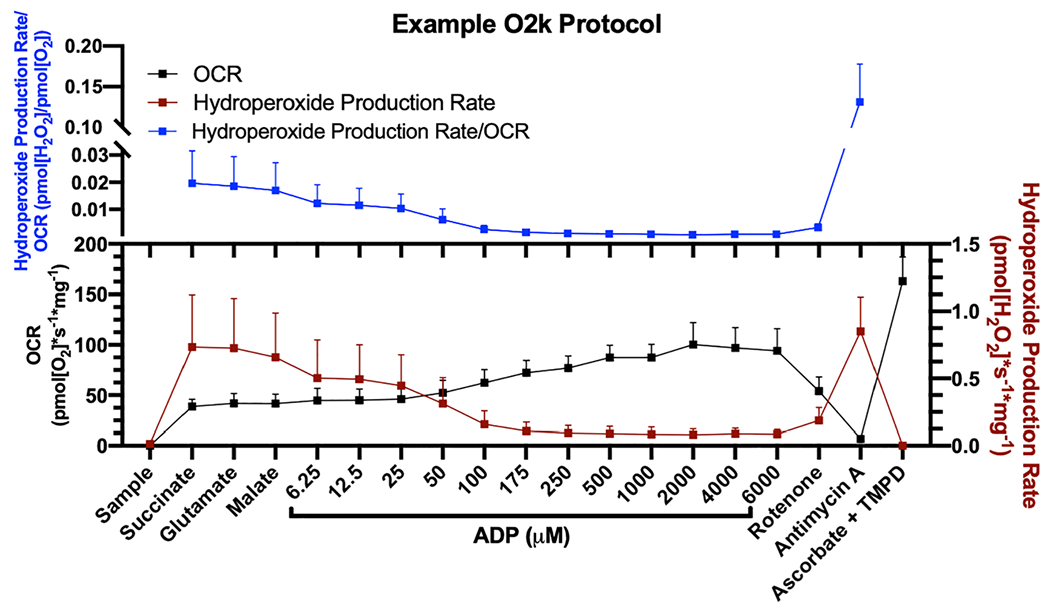
Example O2k SUIT protocol to measure ADP sensitivity. Example protocol in permeabilized gastrocnemius fibres from 6 month wildtype male mice (*n* = 6) showing the order of substrate and inhibitor additions, ADP titration concentrations, and how these substrates affect OCR, hydroperoxide production rate, and the amount of hydroperoxides produced per oxygen consumed. ADP, adenosine diphosphate; OCR, oxygen consumption rate.

We simultaneously measured OCR and ROS production in young, middle-aged, and old permeabilized mouse gastrocnemius fibres. We used a modified version of the protocol described in Holloway *et al*. in which we added succinate, glutamate, and malate as ETS complex I (CI) and complex II (CII) substrates then titrated in ADP to stimulate respiration and inhibit ROS production (Figure 1).^12^ In this protocol, addition of the CI and CII substrates induces reverse electron flow and mitochondrial ETS-derived ROS production that we measure as hydroperoxide production rate with Amplex UltraRed. Addition of superoxide dismutase to the reaction media ensures measurement of both superoxide and hydrogen peroxide produced by the ETS. As ADP is titrated in, the ETS shifts from reverse electron flow to oxidative phosphorylation (OXPHOS). Sensitivity to ADP is measured in the kinetics of when this shift from ROS production to OXPHOS occurs at specified ADP concentrations.

**Figure 2:**
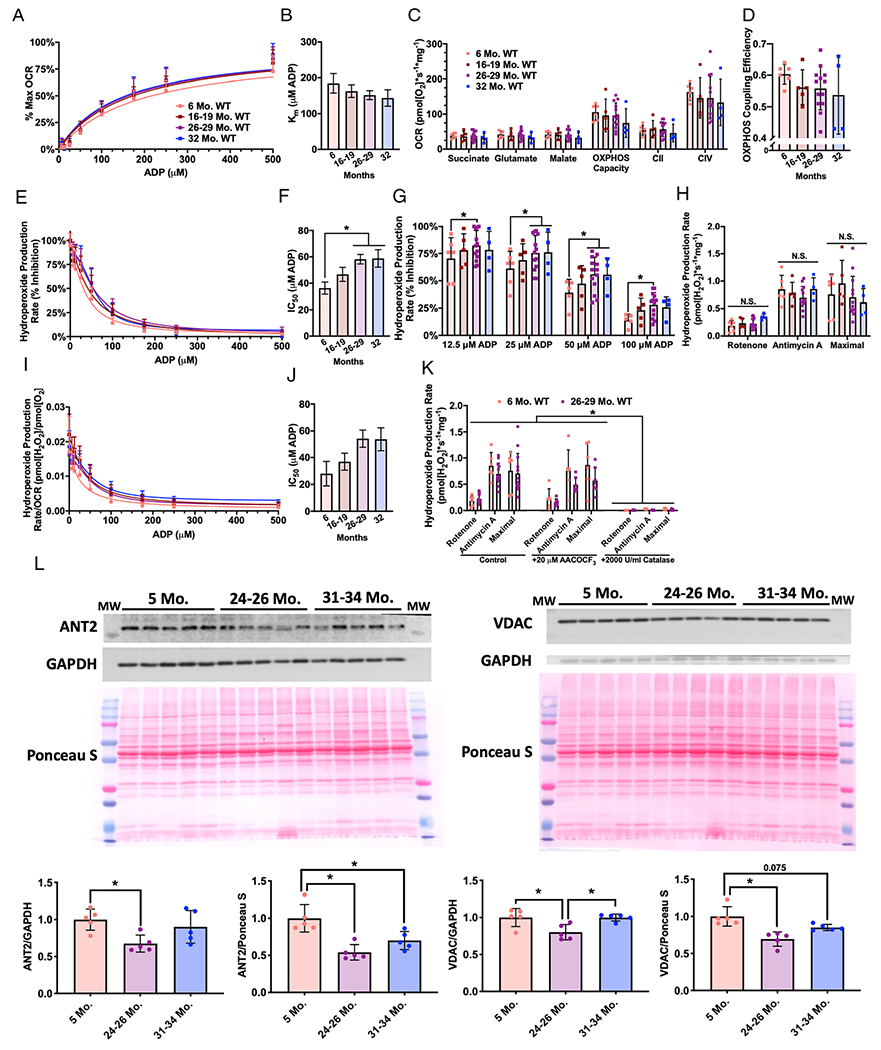
Sensitivity to ADP decreases in aging mouse skeletal muscle. Simultaneous measurement of OCR and hydroperoxide production rates in permeabilized red gastrocnemius fibres from 6 month (pink, *n* = 6 M), 16–19 month (red, *n* = 5 M), 26–29 month (purple, *n* = 4F and 9 M), and 32 month (blue, *n* = 1F and 3 M) wildtype mice. (A) Per cent of maximum OCR during ADP titration and (B) the concentration of ADP required to achieve half of OCR *V*_max_ (Michaelis constant, *K*_m_). (C) OCR for leak respiration states (succinate, glutamate, and malate), OXPHOS capacity (maximum OCR during ADP titration), CII-linked respiration (after addition of the CI inhibitor rotenone), and CIV-linked respiration (after addition of ascorbate and TMPD). (D) OXPHOS coupling efficiency defined as 1-(Leak respiration/OXPHOS capacity), where leak respiration was the OCR after addition of succinate, glutamate, and malate. (E) Hydroperoxide production rate percent inhibition during ADP titration, (F) the concentration of ADP required to achieve 50% inhibition of hydroperoxide production rate (half maximal inhibitory concentration, IC_50_), and (G) the per cent inhibition of hydroperoxide production rate at ADP concentrations with a significant difference. (H) The capability to produce hydroperoxides under conditions stimulated with ETS inhibitors rotenone or antimycin A and maximal hydroperoxide production rate (the highest rate after addition of succinate, glutamate, or malate). (I) Hydroperoxide production rate per OCR during ADP titration and (J) the concentration of ADP required to achieve 50% inhibition (half maximal inhibitory concentration, IC_50_). (K) The capability to produce hydroperoxides under maximal conditions or stimulated with ETS inhibitors after treatment with vehicle, AACOCF_3_, or catalase [6 month (pink, *n* = 6 M) and 26–29 month (purple, *n* = 4F and 9 M)]. (L) Western blots and quantification for ANT2, VDAC, glyceraldehyde 3-phosphate dehydrogenase (GAPDH), and Ponceau S. *P < 0.05 for comparison. N.S., non-significant. ADP, adenosine diphosphate; OCR, oxygen consumption rate.

We find no age-related change in ADP sensitivity kinetics for OCR (Figure 2A–2B). Furthermore, there is no change in leak respiration (succinate, glutamate, or malate), OXPHOS capacity, CII-linked respiration, CIV-linked respiration, or OXPHOS coupling efficiency with this protocol (Figure 2C–2D). The mouse gastrocnemius has an age-related decrease in mitochondrial ADP sensitivity regarding ROS production (Figure 2E–2G). The amount of ADP required to decrease ROS production increases with age (62%), and aged gastrocnemius fibres produce more ROS at physiological ADP concentrations (Figure 2F–2G). However, neither the maximum capacity to produce ROS (measured after addition of succinate, glutamate, and malate without ADP) or the ROS production measured in the presence of the ETS inhibitors rotenone and antimycin A increases or decreases with age (Figure 2H). The amount of ROS produced per oxygen consumed has a trend to increase with age [^~^92–94% increase compared with young, *P* = 0.07 for analysis of variance (ANOVA)] (Figure 2I–2J).

Amplex UltraRed reacts with hydroperoxides, which includes both hydrogen peroxide and lipid hydroperoxides, to produce the fluorescent compound resorufin.^21^ We previously reported that loss of innervation to muscle fibres can induce lipid hydroperoxides produced in the cytosolic phospholipase A_2_ (cPLA_2_) pathway.^21^ To determine whether the hydroperoxides we were measuring were composed of superoxide and hydrogen peroxide (likely from the mitochondrial ETS) or lipid hydroperoxides, we measured hydroperoxide production rates from young and old permeabilized gastrocnemius fibres in vehicle conditions, after treatment with the cPLA_2_ inhibitor AACOCF_3_, or in the presence of the hydrogen peroxide scavenger catalase (Figure 2K). We find that under all three conditions, the hydroperoxides we are measuring are completely inhibited by catalase but not by AACOCF_3_; therefore, these conditions induce production of superoxide and hydrogen peroxide and not lipid hydroperoxides (Figure 2K).

We measured protein content of ADP/ATP translocase 2 (ANT2) and the voltage-dependent anion channel (VDAC) to assess whether there are changes in the expression of these proteins that regulate flux of ADP, ATP, and other metabolites through the mitochondrial membranes. ANT2 and VDAC protein content is decreased in gastrocnemius homogenate from aged mice (24–26 months) compared with young mice (Figure 2I).

### Denervation does not induce adenosine diphosphate insensitivity

**Figure 3:**
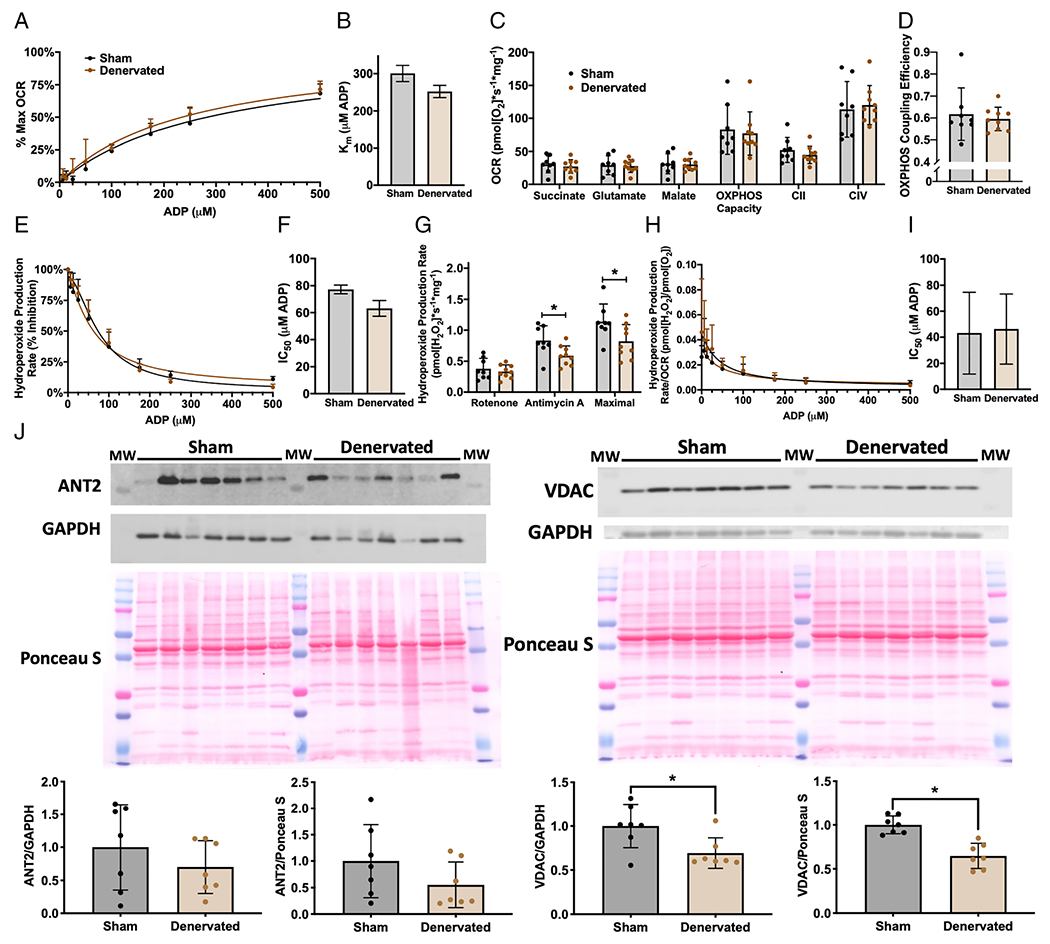
Denervation does not induce ADP insensitivity. Simultaneous measurement of OCR and hydroperoxide production rates in permeabilized red gastrocnemius fibres from 6-month-old to 8-month-old male wildtype mice 7 days after sham (black, *n* = 8) and sciatic nerve transection (brown, *n* = 9) surgeries. (A) Per cent of maximum OCR during ADP titration and (B) the concentration of ADP required to achieve half of OCR *V*_max_ (Michaelis constant, *K*_m_). (C) OCR for leak respiration states (succinate, glutamate, and malate), OXPHOS capacity (maximum OCR during ADP titration), CII-linked respiration (after addition of the CI inhibitor rotenone), and CIV-linked respiration (after addition of ascorbate and TMPD). (D) OXPHOS coupling efficiency defined as 1-(Leak respiration/OXPHOS capacity), where leak respiration was the OCR after addition of succinate, glutamate, and malate. (E) Hydroperoxide production rate per cent inhibition during ADP titration and (F) the concentration of ADP required to achieve 50% inhibition of hydroperoxide production rate (half maximal inhibitory concentration, IC_50_). (G) The capability to produce hydroperoxides under conditions stimulated with ETS inhibitors rotenone or antimycin A and maximal hydroperoxide production rate (the highest rate after addition of succinate, glutamate, or malate). (H) Hydroperoxide production rate per OCR during ADP titration and (I) the concentration of ADP required to achieve 50% inhibition (half maximal inhibitory concentration, IC_50_). (J) Western blots and quantification for ANT2, VDAC, GAPDH, and Ponceau S. *P < 0.05 for comparison.

Loss of neuromuscular innervation increases with age and directly results in muscle atrophy.^22–24^ We used surgical denervation as a model to test whether loss of innervation can induce ADP insensitivity. We performed sciatic nerve transection and sham surgeries on contralateral hindlimbs and then measured ADP sensitivity in sham and denervated permeabilized gastrocnemius fibres 7 days after surgery. Surgical denervation results in progressive muscle atrophy and increased basal hydroperoxide production (in the absence of any substrates or inhibitors) from isolated mitochondria beginning by 7 days, although we have previously shown these hydroperoxides are primarily lipid hydroperoxides.^17,21^ Loss of innervation does not significantly affect OCR kinetics (Figure 3A–3B). There is also no change in leak respiration (succinate, glutamate, or malate), OXPHOS capacity, CII-linked respiration, CIV-linked respiration, or OXPHOS coupling efficiency (Figure 3C–3D). There is no difference in ADP sensitivity kinetics for ROS production; however, loss of innervation does surprisingly decrease the ability of permeabilized fibres to produce hydroperoxides after addition of antimycin A and during maximal stimulation conditions (Figure 3E–3G). Denervated fibres also show no difference in ROS produced per oxygen consumed (Figure 3H–3I). Although VDAC protein content is significantly decreased, there is no significant difference in ANT2 in sham and denervated gastrocnemius homogenate (Figure 2J). Together, these data show that loss of innervation does not induce mitochondrial ADP insensitivity.

### SS-31 treatment improves adenosine diphosphate sensitivity and muscle fatigue resistance in *Sod1*^−/−^ mice

**Figure 4:**
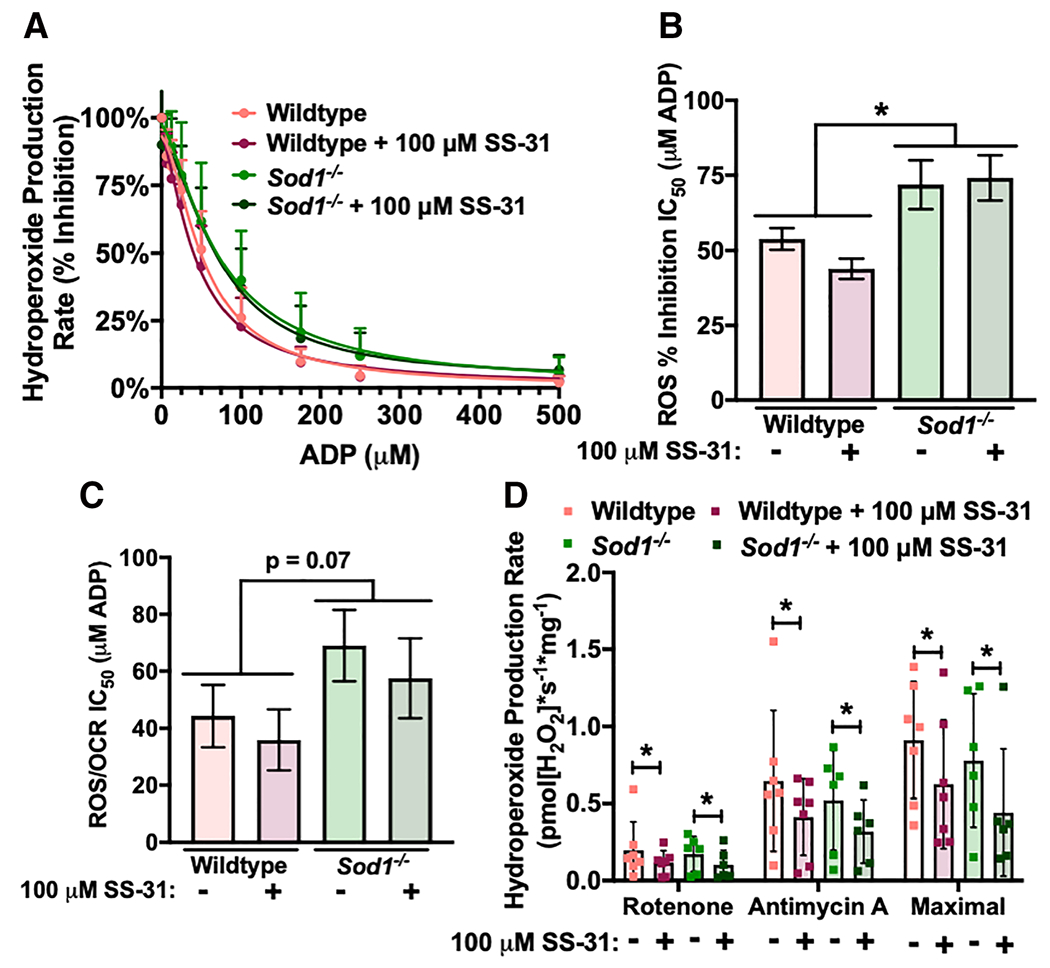
*Sod1*^−/−^ mice are insensitive to ADP, and *in vitro* SS-31 treatment decreases ROS production. Measurement of hydroperoxide production rates in permeabilized red gastrocnemius fibres from 8-month-old to 9-month-old female wildtype (*n* = 7) and *Sod1*^−/−^ (*n* = 6) mice after treatment with vehicle or 100 μM of SS-31 during fibre preparation and assay conditions. Vehicle-treated wildtype (pink), SS-31-treated wildtype (red), vehicle-treated *Sod1*^−/−^ (light green), and SS-31-treated *Sod1*^−/−^ (dark green). (A) Hydroperoxide production rate per cent inhibition during ADP titration and (B) the concentration of ADP required to achieve 50% inhibition of hydroperoxide production rate (half maximal inhibitory concentration, IC_50_). (C) The concentration of ADP required to achieve 50% inhibition (half maximal inhibitory concentration, IC_50_) for hydroperoxide production rate per OCR during ADP titration. (D) The capability to produce hydroperoxides under conditions stimulated with ETS inhibitors rotenone or antimycin A and maximal hydroperoxide production rate (the highest rate after addition of succinate, glutamate, or malate). *P < 0.05 for comparison. ADP, adenosine diphosphate; OCR, oxygen consumption rate.

*Sod1*^−/−^ mice lack the antioxidant enzyme CuZn superoxide dismutase, which scavenges superoxide in the cytosol. We have previously shown that the increased oxidative stress in this model causes early onset sarcopenia characterized by a breakdown in neuromuscular innervation, muscle atrophy, and muscle mitochondrial dysfunction.^16^ Gastrocnemius fibres from 8-month-old to 9-month-old *Sod1*^−/−^ mice are insensitive to ADP compared with wildtype controls and produce more ROS at physiological ADP concentrations (Figure 4A–4B). There is also a trend (ANOVA *P* = 0.07 for genotype) for increased IC_50_ in hydroperoxides produced per oxygen consumed in *Sod1*^−/−^ mice (Figure 4C). However, permeabilized fibres from *Sod1*^−/−^ mice do not produce increased ROS under stimulated conditions (Figure 4D).

SS-31 is a mitochondrial-targeted peptide that repairs mitochondrial structure in several disease states to a normal, healthy structure through cardiolipin and OXPHOS protein interactions and can decrease ROS generation.^25–27^ We added a high dose of SS-31 (100 μM) to permeabilized gastrocnemius fibres from wildtype and *Sod1*^−/−^ mice *ex vivo* during fibre preparation and during the assay to measure whether an acute dose could affect ADP sensitivity. An acute dose of SS-31 does not have an effect on ADP sensitivity; however, it decreases muscle ROS production under stimulated conditions in both wildtype and *Sod1*^−/−^ mice (ANOVA *P* < 0.05 for SS-31 treatment) (Figure 4D). There is no difference between wildtype and *Sod1*^−/−^ mice with or without SS-31 treatment for OCR ADP sensitivity kinetics, leak respiration (succinate, glutamate, or malate), OXPHOS capacity, CII-linked respiration, CIV-linked respiration, or OXPHOS coupling efficiency (data not shown).

**Figure 5:**
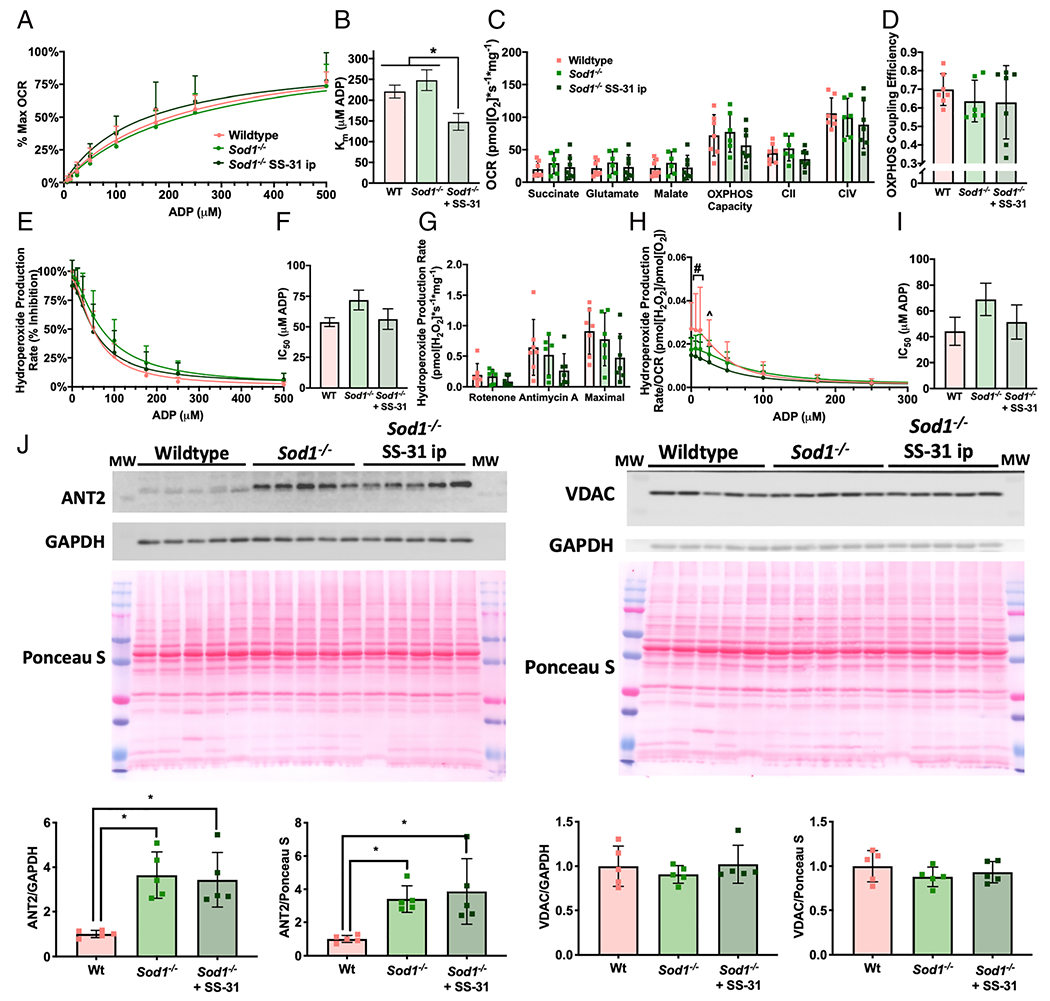
Ten days of SS-31 treatment increases ADP sensitivity in *Sod1*^−/−^ mice. Simultaneous measurement of OCR and hydroperoxide production rates in permeabilized red gastrocnemius fibres from 8-month-old to 9-month-old female wildtype and *Sod1*^−/−^ mice after daily ip injection of saline or 10 mg/kg SS-31. Saline-injected wildtype (pink, *n* = 7), saline-injected *Sod1*^−/−^ (light green, *n* = 6), and SS-31-injected *Sod1*^−/−^ (dark green, *n* = 8). (A) Per cent of maximum OCR during ADP titration and (B) the concentration of ADP required to achieve half of OCR *V*_max_ (Michaelis constant, *K*_m_). (C) OCR for leak respiration states (succinate, glutamate, and malate), OXPHOS capacity (maximum OCR during ADP titration), CII-linked respiration (after addition of the CI inhibitor rotenone), and CIV-linked respiration (after addition of ascorbate and TMPD). (D) OXPHOS coupling efficiency defined as 1-(Leak respiration/OXPHOS capacity), where leak respiration was the OCR after addition of succinate, glutamate, and malate. (E) Hydroperoxide production rate per cent inhibition during ADP titration and (F) the concentration of ADP required to achieve 50% inhibition of hydroperoxide production rate (half maximal inhibitory concentration, IC_50_). (G) The capability to produce hydroperoxides under conditions stimulated with ETS inhibitors rotenone or antimycin A and maximal hydroperoxide production rate (the highest rate after addition of succinate, glutamate, or malate). (H) Hydroperoxide production rate per OCR during ADP titration and (I) the concentration of ADP required to achieve 50% inhibition (half maximal inhibitory concentration, IC_50_). (J) Western blots and quantification for ANT2, VDAC, GAPDH, and Ponceau S. *P < 0.05 for comparison. ^#^*P* < 0.05 saline-treated wildtype vs. saline-treated and SS-31-treated *Sod1*^−/−^. ^^^*P* < 0.05 saline-treated wildtype vs. saline-treated *Sod1*^−/−^. ADP, adenosine diphosphate; OCR, oxygen consumption rate.

To determine the *in vivo* effects of SS-31 treatment, we treated *Sod1*^−/−^ mice with SS-31 once per day (ip) and wildtype and *Sod1*^−/−^ mice with saline injections once per day (vehicle control) for 10 days. After 10 days of treatment, we measured ADP sensitivity in permeabilized gastrocnemius fibres and *ex vivo* contractile force in the extensor digitorum longus (EDL) muscle. SS-31 treatment increased sensitivity to ADP for OCR kinetics in the *Sod1*^−/−^ mice, while it does not affect leak respiration (succinate, glutamate, or malate), OXPHOS capacity, CII-linked respiration, CIV-linked respiration, or OXPHOS coupling efficiency (Figure 5A–5D). *In vivo* SS-31 treatment blunted the ROS production due to ADP insensitivity, although this affect did not reach statistical significance in this experiment. (Figure 5E–5F). SS-31 treatment decreased stimulated ROS in *Sod1*^−/−^ mice when conditions were analysed together (two-way ANOVA *P* < 0.05 for genotype/treatment) (Figure 5G). Surprisingly, wildtype mice produced more ROS per oxygen consumed at low ADP concentrations than vehicle-treated or SS-31-treated *Sod1*^−/−^ mice (Figure 5H). Although it did not reach statistical significance, IC_50_ for hydroperoxides produced per oxygen consumed is increased in vehicle-treated *Sod1*^−/−^ mice (56%) and decreased to near wildtype levels by SS-31 treatment (Figure 5I). ANT2 protein content is significantly increased in the gastrocnemius muscle from *Sod1*^−/−^ mice (^~^240–264%), while there is no statistically significant difference in VDAC (Figure 2J). We observed no difference in ANT2 or VDAC protein content in *Sod1*^−/−^ mice following 10 days of SS-31 treatment.

**Figure 6:**
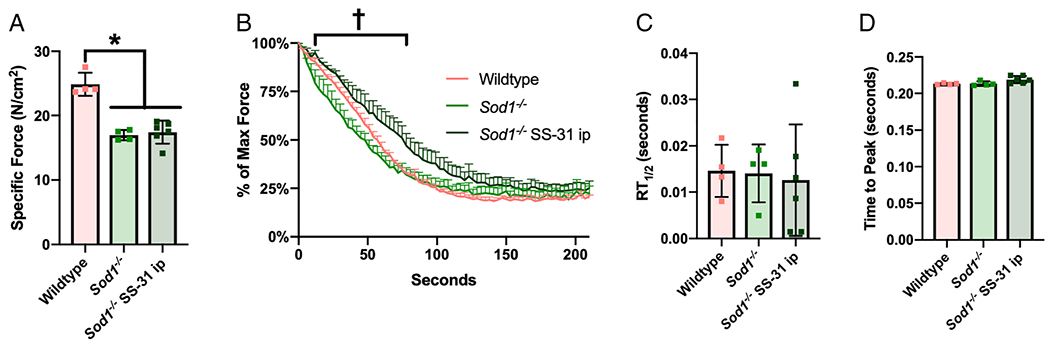
SS-31 treatment improves muscle fatigue resistance in *Sod1*^−/−^ mice. Contractile properties of isolated EDL muscles from 8-month-old to 9-month-old female wildtype and *Sod1*^−/−^ mice after daily ip injection of saline or 10 mg/kg of SS-31. Saline-injected wildtype (pink, *n* = 4), saline-injected *Sod1*^−/−^ (light green, *n* = 4), and SS-31-injected *Sod1*^−/−^ (dark green, *n* = 6). EDL muscle (A) specific force (N/cm^2^) measured using muscle length and mass, (B) fatigue resistance as per cent of maximum force during a sustained fatigue protocol, (C) time to reach half relaxation from the peak (RT_1/2_), and (D) the time to peak (TTP) force. *P < 0.05 for comparison. ^†^*P* < 0.05 saline-treated *Sod1*^−/−^ vs. SS-31-treated *Sod1*^−/−^.

*Sod1*^−/−^ mice have decreased specific force in the EDL muscle compared with wildtype mice, and *in vivo* SS-31 treatment does not affect this phenotype (Figure 6A). However, SS-31 treatment improves resistance to fatigue during sustained contractions in *Sod1*^−/−^ mice (Figure 6B). No difference was observed in half-relaxation time (RT_1/2_); however, SS-31 treatment slightly increased time to peak force (ANOVA *P* = 0.052) (Figure 6C–6D). Hindlimb muscle mass was decreased in *Sod1*^−/−^ mice as previously reported and SS-31 treatment does not affect muscle mass after 10 days (data not shown).^16^

## Discussion

### Adenosine diphosphate sensitivity decreases in human and mouse skeletal muscle with age

Holloway *et al*. recently reported a decrease in mitochondrial ADP sensitivity in aged human *vastus lateralis* biopsies.^12^ Here, we show that aged mouse gastrocnemius muscles also experience a decrease in mitochondrial ADP sensitivity that results in increased mitochondrial ROS production at physiological ADP concentrations. There is also no significant change in OXPHOS capacity or maximal ROS production observed in aged mice using the same protocol, which is consistent with previous reports that OXPHOS capacity and maximal ROS production do not change with age in human muscle.^7,8,12^ In human muscle, ADP insensitivity also results in an increase in ROS produced per oxygen consumed at physiological ADP concentrations.^12^ We found a trend for this increase in fibres from aged mice, although it did not reach statistical significance. There are several differences in the ADP insensitivity phenotype between aged muscle from mice and humans. ADP insensitivity in human muscle results in a decrease in the ADP titration OCR *K*_m_ that we do not observe in mice.^12^ We also find that muscle from mice was more sensitive to low concentrations of ADP compared with the reported values for humans. We modified our protocol to include more concentrations at low ADP, because the IC_50_ for ROS production inhibition in aged mice was approximately one-thirds of that reported in senescent human fibres.^12^

### Potential mechanisms for adenosine diphosphate insensitivity

The mechanism of age-related insensitivity to ADP is still unclear, although the potential causes included loss of innervation, changes in ADP and substrate transport, changes in OXPHOS components, increased oxidative stress and decreased redox capabilities, and age-related changes in mitochondrial protein interactions, structure, and networks. Furthermore, the age-related decline in muscle strength and fatigue resistance cannot be fully explained by the decline in muscle mass, and ADP insensitivity provides a potential explanation for decreased muscle strength. Elucidating the mechanism of ADP insensitivity will allow the development of targeted therapeutics and could reveal whether it plays a role in the decline of muscle mass or contractile function. We next describe the literature on each of these mechanisms in detail.

### Loss of innervation induces lipid hydroperoxide production but not adenosine diphosphate insensitivity

Breakdown in neuromuscular innervation increases with age and is a driving factor in the decline in muscle mass.^22–24^ Our laboratory has identified two separate age-related phenotypes of increased hydroperoxide production in aging muscle: (i) increased lipid hydroperoxide production from arachidonic acid released by cPLA_2_ that is induced by cPLA_2_ activation after loss of innervation (including surgical denervation, genetic, and aging models) and (ii) the experiments described here showing an age-related increase in production of superoxide and hydrogen peroxide by the mitochondrial ETS at physiological concentrations of ADP.^17,21,28,29^ Denervated muscle fibres produce increased lipid hydroperoxides under basal conditions (hydroperoxide production in the absence of mitochondrial substrates or ADP) in several models including surgical, genetic models, and aging models.^17,21,28,30^ The increase in basal hydroperoxide production due to denervation is caused primarily by increased production of lipid hydroperoxides from arachidonic acid via cPLA_2_.^17,21,28,30^ Critically, under basal conditions, there are no substrates entering the mitochondrial ETS or ADP to drive production of oxygen radicals by electrons in the system.

Whether loss of innervation also contributes to ADP insensitivity is currently unknown. In this report, we measure ADP insensitivity in denervated muscle fibres by adding substrates for the ETS and titrating in ADP. We report here that loss of innervation is not sufficient to induce ADP insensitivity. Furthermore, the trend for the denervated samples is for the denervated fibres to actually be slightly more sensitive to ADP than sham, although overall, there is no statistically significant effect. The magnitude of change was much larger in the aging and *Sod1*^−/−^ experiments as well, that is IC_50_ for hydroperoxide production rate (% inhibition) increases 62% in aging and 33% in 8-month-old to 9-month-old *Sod1*^−/−^ mouse gastrocnemius, while the decrease of 18% in denervated vs. sham gastrocnemius is not significant. Furthermore, we identify that ADP insensitivity is composed entirely of increased superoxide and hydrogen peroxide production and not by increased lipid hydroperoxides (Figure 2K).

### Protein content changes in mitochondrial adenosine diphosphate and substrate transporters in adenosine diphosphate insensitivity

Altered levels or activity of ADP and substrate transportation proteins are a potential mechanism for ADP insensitivity as there is a reduction in VDAC content in aged human *vastus lateralis*.^12^ Supporting this mechanism, *Vdac1*^−/−^ mice have decreased sensitivity to ADP in permeabilized gastrocnemius fibres.^31^ However, ANT protein content did not differ between young and aged human muscle samples that showed reduced ADP sensitivity.^12^ In the current study, we found a decrease in VDAC and ANT2 protein content in aged mouse gastrocnemius muscles; however, we previously found no difference in ANT1 protein content between young and aged gastrocnemius muscles using a targeted proteomics approach.^32^ Further, data from denervated muscle and *Sod1*^−/−^ muscle provide evidence against changes in VDAC and ANT2 protein content explaining the loss in sensitivity to ADP, as denervated muscle fibres did not exhibit ADP insensitivity despite a decrease in VDAC protein content. *Sod1*^−/−^ mice also exhibit ADP insensitivity while having greatly increased ANT2 protein content and no difference in VDAC protein content. Furthermore, treatment with SS-31 improved ADP sensitivity in *Sod1*^−/−^ mice without affecting protein content of ANT2 or VDAC, suggesting the changes in VDAC and ANT protein content observed in aged mice may not underlie or completely explain the ADP insensitivity phenotype.

### Protein content changes in oxidative phosphorylation components are not linked to adenosine diphosphate insensitivity

We have previously reported the abundance of several OXPHOS complex proteins as well as other enzymes involved in oxidative metabolism. In Walsh *et al.*, we used targeted-proteomics to measure protein content in adult (12 months) and aged (26 months) mouse skeletal muscle including enzymes involved in oxidative metabolism.^30^ In Ahn *et al.*, we also performed targeted proteomics in young (4 months) and aged (24 months) mouse gastrocnemius muscles with a more detailed focus on oxidative metabolism components.^32^ Most critically, the succinate-driven respirometry protocol applied in this manuscript introduces electrons to the electron transport chain through succinate dehydrogenase/complex II that produce ROS primarily by reverse electron flow through site Complex I_Q_.^33^ In both Walsh *et al.* and Ahn *et al*., we report no change in the subunits of succinate dehydrogenase (SDHA, SDHB, and SDHC) in protein content between young and old mouse skeletal muscle. In Ahn *et al*., we analysed additional ETS proteins and report no difference for protein content of all measured proteins including Complex I subunits (NDUFS1, NDUFV1), Electron Transfer Flavoprotein subunits (ETFA, ETFB, ETFDH), Complex III subunit (UQCRC1), Coenzyme Q6 monooxygenase, or Complex V/ATP Synthase (ATP5A1, ATP5B). These findings suggest that changes in levels of proteins supporting OXPHOS are not a primary contributor to the ADP insensitivity phenotype we report here.

### Oxidative stress contributes to adenosine diphosphate insensitivity

One hypothesis to explain the age-related changes in ADP insensitivity is decreased redox capability and increased oxidative stress and damage. Redox maintenance decreases and oxidative stress increases in aged skeletal muscle from both mice and humans.^12,32^ Protein content and enzyme activity for antioxidants related to superoxide and hydrogen peroxide scavenging does not change or increases with age in skeletal muscle. For antioxidant protein content, in Walsh *et al*., we observed an increase in CuZnSOD protein content with age (26 months) compared with adult (12 months) mice, and we did not observe a statistically significant change with age for MnSOD, Catalase, or GPX1 compared with adult mice.^30^ In Ahn *et al*., we observed an increase in GPX1 protein content with age (24 months) compared with young (4 months) mice, and we did not observe a statistically significant change with age for MnSOD or CuZnSOD protein content.^32^ The disparity in results underscores the difficulties in assigning causality to changes in antioxidant protein levels to mechanistic changes. The levels are highly variable and rarely consistent in our experience. For antioxidant enzyme activity, in Walsh *et al*., we did not observe a change in age for catalase, MnSOD, or CuZnSOD, but we did observe an age-related increase in GPX enzyme activity.^30^ However, we also observe an increase in oxidative damage and change in the oxidative status of the muscle. We have demonstrated an age-related increase in muscle oxidative damage including increased protein carbonyls, increased F_2_-isoprostanes, and decreased GSH : GSSG ratio with age.^15,30,32^ We propose throughout our work that changes in levels of proteins involved in antioxidant defence system is insufficient to counteract increased oxidative stress with age.^32^

We identify ADP insensitivity as a potential phenotype driving increased ROS production in physiological conditions that contributes to the observed age-related increase in oxidative damage. The *Sod1*^−/−^ mouse model develops accelerated sarcopenia including high levels of oxidative stress and damage by 5 months of age.^18^ We report that *Sod1*^−/−^ mice also experience ADP insensitivity by 8–9 months of age, which provides some evidential support to the oxidative stress hypothesis.

### Changes in mitochondrial protein interactions, structure, and networks

Changes in mitochondrial protein interactions, cristae structure, and networks may also play a role in ADP insensitivity. The cristae structure of mitochondria is critical to ETS efficiency.^25,34^ Protein–protein interaction of mitochondrial proteins plays a critical role in energy production, and how these interactions change with age is poorly understood.^34^ The content of cardiolipin, an inner mitochondrial membrane phospholipid, decreases in skeletal muscle mitochondria with age, which decreases the folding of mitochondrial cristae and decreases ETS efficiency.^25,35^ Mitochondria also form complex interconnected networks in skeletal muscle, which are critical to efficient energy production and distribution, and these networks have been found to have specific localization of ETS complexes and ATP synthases in discreet parts of the cell.^36^ How these mitochondrial networks and their localization of proteins that produce vs. use the proton motive force change with age is not understood, but it provides another avenue that could contribute to mitochondrial ADP insensitivity.

### Development of interventions to target adenosine diphosphate insensitivity

Although they could not elucidate the mechanism, Holloway *et al*. also used resistance training as an intervention to target ADP insensitivity in aged individuals.^12^ Resistance training increased muscle mass, strength, and mitochondrial OXPHOS capacity as expected, but it was also able to partially target ADP insensitivity.^12^ Resistance training improved the *K*_m_ for OCR and IC_50_ for ROS production in aged individuals, although it does not affect absolute ROS production at physiological ADP concentrations, ROS produced per oxygen consumed, or content of ADP transporters, which also suggests the decline in VDAC may be unrelated to the mechanism of ADP insensitivity.^12^

Previous studies demonstrate that the tetrapeptide SS-31 exerts multifaceted mitochondrial protective effects, including attenuation of mitochondrial ROS production, preservation of mitochondrial cristae structure, improvement of ETS efficiency, and restoration of mitochondrial respiration and cellular bioenergetics in mouse models of aging and accelerated aging (e.g. high-fat diet feeding).^19,20,25^ We report here that SS-31 increases mitochondrial sensitivity to ADP for OCR, decreases stimulated ROS production capabilities, and increases resistance to muscle fatigue in a mouse model of oxidative stress-induced sarcopenia. This experiment carries the caveat that we measured ADP sensitivity in gastrocnemius fibres and not EDL fibres. However, these findings agree with the conclusions of previous investigations showing beneficial effects of SS-31 treatment on muscle bioenergetics and contractile properties. An acute (1 h) treatment with SS-31 was previously shown to restore muscle mitochondrial bioenergetics and increase fatigue resistance but not specific force in aged mice.^20^ A 100 μM of exogenous SS-31 also decreased succinate-stimulated ROS production in EDL fibres from aged mice, which is similar to the results we obtained for maximal (succinate-stimulated) ROS production in *Sod1*^−/−^ gastrocnemius fibres.^20^ Eight-week treatment of old mice with SS-31 also repaired mitochondrial bioenergetics and improved muscle fatigue in the gastrocnemius muscle.^19^ SS-31 increases sensitivity to ADP *in vivo* in aged muscle mitochondria.^27^ Interestingly, these authors also found that ATP concentrations in the tibialis anterior muscle were decreased and *in vivo* ADP concentrations determined from ^31^P magnetic resonance spectroscopy for the distal hindlimb were increased sixfold to sevenfold with age, while SS-31 treatment was able to partially repair these age-related changes in metabolite concentrations.^19^

These studies, in conjunction with the results reported here, provide evidence for SS-31 as the first pharmacological intervention that can target mitochondrial ADP insensitivity in skeletal muscle and suggest that ADP insensitivity contributes to muscle fatigue. How SS-31 targets ADP insensitivity is not yet completely understood. SS-31 was recently reported to bind to proteins present in ETS complexes III and IV as well as proteins involved in ADP/ATP transport and production including ATP synthase, creatine kinase, and ANT1.^27^ In addition to binding to cardiolipin and remodelling mitochondrial cristae structure, these targets of SS-31 represent attractive mechanisms.^25–27^ Importantly, SS-31 is already in clinical trials for use in patients with mitochondrial myopathy (ClinicalTrials.gov Identifier: NCT03098797) and age-related macular degeneration due to mitochondrial dysfunction (ClinicalTrials.gov Identifier: NCT03891875). Thus, the development of SS-31-based therapies for mitochondrial protection and prevention of sarcopenia in older adults remains an attractive possibility.

## Conclusions

Adenosine diphosphate insensitivity in aged mouse and human skeletal muscle is associated with increased ROS production in both species and decreased OCR production in humans.^12^ Loss of neuromuscular innervation is not sufficient to induce ADP sensitivity in the mouse gastrocnemius muscle, while a mouse model of increased oxidative stress exhibited ADP insensitivity. A clear mechanistic understanding for the cause of ADP insensitivity remains elusive, but increased oxidative stress and age-related changes in mitochondrial structure and networks appear as key contenders. Decreased sensitivity to ADP may contribute to muscle fatigue, and we report here that SS-31 treatment targets both ADP insensitivity and muscle fatigue.

### Remaining questions and future directions

Beyond mechanism, other key questions remain about age-related ADP insensitivity. Do other tissues and cell types experience age-related ADP insensitivity? We do not know whether ADP insensitivity is common to all muscles with age or whether it is driven by changes in certain fibre types that are more abundant in certain muscles. Do all species experience ADP insensitivity in aging muscle, and do they experience it the same way? We now know that aged mouse and human skeletal muscle are both insensitive to ADP, although there are differences in the phenotype. What causes skeletal muscle from humans to also be ADP insensitive for the kinetics of OCR? If some species do not experience ADP insensitivity, what is different about how their muscles age? It is also unknown whether ADP insensitivity can affect the decline in muscle mass and strength, although treatment with SS-31 was previously reported to increase muscle mass in aged mice.^19^ Another caveat of these experiments is the use of supraphysiological concentrations of mitochondrial substrates. Future studies should make use of physiological concentrations of muscle substrates such as pyruvate to more closely model *in vivo* conditions. By answering some of these questions, we can identify therapeutic targets and determine the role of ADP insensitivity in the age-related decline of muscle mass, quality, and function.

## Materials and methods

### Animals

The study was approved by the Institutional Animal Care and Use Committees at the University of Oklahoma Health Sciences Center and the Oklahoma Medical Research Foundation. All experiments used mice from strains on a C57BL/6J genetic background currently housed in the mouse colony of Dr Van Remmen at the Oklahoma Medical Research Foundation or Veterans Affairs Medical Center. *Sod1*^−/−^ mice were previously described.^37^ Mice were caged in a pathogen-free environment with free access to standard chow and water and maintained on a 12 h of light/dark cycle. Mice were euthanized using a CO_2_ chamber and cervical dislocation. Hindlimb muscles were dissected and weighed, and fresh biopsies were cut for permeabilized muscle fibre experiments and EDL contractility.

### Muscle fibre permeabilization and simultaneous oxygen consumption rate and hydroperoxide production rate measurement

Permeabilized red gastrocnemius muscle fibre bundles (^~^3–5 mg) were prepared by mechanical separation, saponin permeabilization, and washing as previously described.^32,38^ Description of assay conditions and explanation of methods for simultaneous measurement of OCR using the Oroboros Oxygraph-2k (O2k, OROBOROS Instruments, Innsbruck, Austria) and hydroperoxide production rate using the O2k-Fluo LED2-Module Fluorescence-Sensor Green with Amplex UltraRed were previously described.^32,38^ The assay chambers were hyper-oxygenated to ^~^400 nmol of O_2_/mL before the assay using injection of oxygen to prevent low oxygen concentrations during the course of the protocol. The substrate–uncoupler–inhibitor titration protocol was based on that described in Holloway *et al*. with modifications including lower ADP concentrations following an increased sensitivity to ADP we observed in mouse fibres compared with reported ADP sensitivity in human fibres.^12^ We used sequential additions of substrates and inhibitors as follows: succinate (10 mM), glutamate (10 mM), malate (0.5 mM), ADP (6.25, 12.5, 25, 50, 100, 175, 250, 500, 1000, 2000, 4000, and 6000 μM), 0.5 μM of rotenone, 5 μM of antimycin A, and simultaneous injection of ascorbate (2 mM) and TMPD (0.5 mM) (Figure 1).

Leak respiration measurements were determined after addition of succinate, glutamate, or malate. OXPHOS capacity was determined as the highest OCR measured during the ADP titrations. CII-linked respiration was measured after addition of rotenone to inhibit CI and to induce ROS production. CIV-linked respiration was measured after addition of ascorbate and TMPD. We added antimycin A to further induce ROS production and measure non-mitochondrial respiration. All OCR measurements were normalized using antimycin A to account for non-mitochondrial OCR. OXPHOS coupling efficiency was calculated as 1-(Leak respiration/OXPHOS capacity), where leak respiration was the OCR after addition of succinate, glutamate, and malate. For per cent max OCR during the ADP titration, the lowest OCR value was set at 0%, and the highest OCR value was set at 100% for each sample including the OCR measurement after addition of malate as 0 μM of ADP.

The hydroperoxide production rate is measured as the change in fluorescence intensity due to resorufin production rate. Maximal hydroperoxide production rate is the highest rate measured after addition of succinate, glutamate, or malate, as there was some variation per sample between each measurement. For hydroperoxide production rate per cent inhibition during the ADP titration, the lowest hydroperoxide production rate value was set at 0%, and the highest hydroperoxide production rate value was set at 100% for each sample including the measurement after addition of malate as 0 μM of ADP. For hydroperoxide production rate, auto-oxidation of Amplex UltraRed is accounted for in all samples by subtracting the background rate from experimental measurements. The ratio of hydroperoxide production rate per OCR is a metric of electron transport efficiency. A higher ratio suggests increased flow of electrons from the ETS to production of ROS instead of energy. All OCR and hydroperoxide production rate measurements were normalized to muscle bundle wet weights.

For the aging study, simultaneous measurement of OCR and hydroperoxide production rates was performed in permeabilized red gastrocnemius fibres from 6 month (*n* = 6 M), 16–19 month (*n* = 5 M), 26–29 month (*n* = 4F and 9 M), and 32 month (*n* = 1F and 3 M) wildtype mice. Permeabilized fibres from young (6 month) and old (26–29 month) C57BL/6 J mice were treated with vehicle, 2000 U/mL of catalase (Sigma), or 20 μM of arachidonyl trifluoromethyl ketone (AACOCF_3_; Cayman Chemicals, Ann Arbor, MI). Catalase treatment occurred during the 30 min of saponin permeabilization, 3 × 5 min wash steps, and in the O2k chamber during the assay. AACOCF_3_ treatment occurred during the 30 min of saponin permeabilization and 3 × 5 min wash steps, but not in the O2k chamber during the assay. A 8-month-old to 9-month-old female C57BL/6J (*n* = 7) and *Sod1*^−/−^ (*n* = 6) permeabilized fibres were treated with 100 μM SS-31 during the 30 min saponin permeabilization, 3 × 5 min wash steps, and in the O2k chamber during the assay. Our reaction conditions contain 5 U/mL of superoxide dismutase, which converts any superoxide produced into hydrogen peroxide.

### Sciatic nerve transection surgeries

Sciatic nerve transection and sham surgeries were performed as previously described on 6-month-old to 8-month-old C57BL/6J male mice.^17,21,28^ The mice were anaesthetised with isoflurane then maintained under anaesthesia using a mouse nose cone on a heated pad. The lateral thigh and buttock from the sciatic notch to the knee were shaved, and 100% ethanol and 2% chlorhexidine were used to clean the skin surface. A small incision was made from the sciatic notch to the knee (^~^1 cm), and the sciatic nerve was exposed. The nerve was cut and a ^~^5 mm piece removed. The ends of the nerve were folded back and sutured to prevent nerve regrowth. On the other leg, the nerve was exposed but not cut and removed. This limb served as a sham-operated control. The incision on each leg was closed using the sutures and skin glue, and the mouse was placed on a warm heating pad until recovered. The mice were treated with subcutaneous injections of 9.5 mg/kg of ketoprofen dissolved in saline once per day for 3 days following surgery to minimize pain. The mice were euthanized and dissected at 7 days after surgery. Simultaneous measurement of OCR and hydroperoxide production rates in permeabilized red gastrocnemius fibres was performed in 6-month-old to 8-month-old male wildtype mice 7 days after sham (*n* = 8) and sciatic nerve transection (*n* = 9) surgeries.

### SS-31 treatment regimen

A cohort of female *Sod1*^−/−^ mice was treated with SS-31 and female C57BL/6J and *Sod1*^−/−^ mice were treated with vehicle control (saline). Mice were treated daily with ip injections of 10 mg/kg of SS-31 or saline for 10 days, then euthanized to collect muscle masses, EDL contractility, and Oroboros O2k OCR and hydroperoxide production rate measurements. Simultaneous measurement of OCR and hydroperoxide production rates was performed in permeabilized red gastrocnemius fibres from 8-month-old to 9-month-old female wildtype and *Sod1*^−/−^ mice after daily ip injection of saline or 10 mg/kg of SS-31. Saline-injected wildtype (*n* = 7), saline-injected *Sod1*^−/−^ (*n* = 6), and SS-31-injected *Sod1*^−/−^ (*n* = 8).

### Extensor digitorum longus contractile properties

Contractile properties of isolated EDL muscle were measured from a cohort of saline wildtype (*n* = 4), saline *Sod1*^−/−^ (*n* = 4), and SS-31-treated *Sod1*^−/−^ (*n* = 6) mice using a 1200A *in vitro* test system (Aurora Scientific Inc., Aurora, ON, Canada) as previously described.^32,39^ EDL muscle was dissected, one end was tied to a hook and the other to a Dual-Mode Muscle Lever System (300C-LR, Aurora Scientific Inc, Aurora, Canada). The muscle was fixed with oxygenated Kreb–Ringer solution at 32°C, and then allowed 20 min to reach thermoequilibration. A model 701C stimulator (Aurora Scientific Inc.) applied muscle stimulation at supramaximal voltage, 0.2 ms of pulse width, and at optimum length (L_o_) for twitch force production. The time to peak force and time to reach half relaxation time from the peak (RT_1/2_) were measured for twitch contractions. Force frequency curves were generated with stimulation frequencies between 1 and 300 Hz. The muscle fatigue protocol consisted of repeated 300 ms trains of 150 Hz stimuli every 3 s for a total of 200 s and is expressed as per cent of the maximum force measured during the fatigue protocol. Specific force (N/cm^2^) was measured using muscle length and mass as previously described.^40^ All data were recorded and analysed using commercial software (DMC and DMA, Aurora Scientific Inc.), Microsoft Office Excel, and GraphPad Prism 7.0b for Mac OS X (GraphPad Software, La Jolla, CA).

### Protein content by western blot

Western blot analysis of protein content was performed as previously described using primary antibodies for ANT2 (1:1,000; Cell Signalling E2B9D), VDAC (1:1,000; Cell Signalling D73D12), and GAPDH (1:10,000; Sigma-Aldrich G9545).^41^ Protein was isolated from gastrocnemius samples for young (5 months old), aged (24–26 months old), and very old (31–34 month old) male mice (*n* = 5), sham and denervated male mice 7 days after sham and sciatic nerve transection surgeries (*n* = 7), and female saline-injected wildtype, saline-injected *Sod1*^−/−^, 10 day SS-31-injected *Sod1*^−/−^ mice (*n* = 5).

### Statistical analyses

The results were analysed using Microsoft Office Excel and GraphPad Prism 7.0b for Mac OS X (GraphPad Software, La Jolla, CA). Non-linear curve fitting and *K*_m_ values were determined for per cent max OCR using Michaelis–Menten non-linear regression with least squares fitting and no weighting in GraphPad Prism. Non-linear curve fitting and IC_50_ values were determined for hydroperoxide production rate per cent inhibition and hydroperoxide production rate per OCR using [inhibitor] vs. response—variable slope (four parameters) with least squares fitting and no weighting in GraphPad Prism. *K*_m_, IC_50_, OCR state measurements, OXPHOS coupling efficiency, hydroperoxide production rate for rotenone, antimycin A, and maximal conditions, and protein content by western blot for aging time course and ip-injected wildtype and *Sod1*^−/−^ mice fibres and specific force, half-relaxation time, and time to peak for wildtype and *Sod1*^−/−^ EDL muscles were compared with ordinary one-way ANOVA with Tukey’s post hoc test. *K*_m_, IC_50_, OCR state measurements, OXPHOS coupling efficiency, hydroperoxide production rate for rotenone, antimycin A, and maximal conditions, and protein content by western blot for sham and denervated fibres were compared with unpaired two-tailed Student’s *t*-test. *K*_m_, IC_50_, OCR state measurements, OXPHOS coupling efficiency, and hydroperoxide production rate for rotenone, antimycin A, and maximal for wildtype and *Sod1*^−/−^ mice treated *ex vivo* with 100 μM of SS-31 were compared with repeated measures two-way ANOVA and Sidak’s post hoc test. Hydroperoxide production rate per cent inhibition at specific ADP concentrations and hydroperoxide production rate with treatment of vehicle, AACOCF_3_, or catalase in the aging study were compared with ordinary two-way ANOVA with Tukey’s post hoc test. Hydroperoxide production rate for rotenone, antimycin A, and maximal for wildtype and *Sod1*^−/−^ mice treated *ex vivo* with 100 μM of SS-31 or with ip SS-31 injections were compared together using two-way ANOVA to determine the effect of SS-31 treatment overall on ROS production. Hydroperoxide production rate per cent inhibition at specific ADP concentrations and hydroperoxide production rate with treatment of vehicle, AACOCF_3_, or catalase in the aging study were compared with ordinary two-way ANOVA with Tukey’s post hoc test. EDL fatigue force was compared with repeated measures two-way ANOVA and Tukey’s post hoc test. For all comparisons, *P* < 0.05 was considered statistically significant. Plots depict mean ± standard deviation, except *K*_m_, IC_50_, and EDL muscle fatigue, which are mean ± standard error of the mean.

## Key Points

- ADP insensitivity is the reduced ability of ADP to stimulate mitochondrial oxygen consumption rate (OCR) or inhibit reactive oxygen species (ROS) production.
- ADP insensitivity in aged mouse skeletal muscle mitochondria parallels increased ROS production.
- ADP insensitivity is associated with increased ROS production in a model of oxidative stress.
- Loss of neuromuscular innervation does not induce ADP insensitivity for either OCR or ROS production.
- SS-31 may be the first drug capable of targeting ADP insensitivity.
- ADP insensitivity could contribute to muscle fatigue.
